# Patients with ischaemic, mixed and nephrotoxic acute tubular necrosis in the intensive care unit – a homogeneous population?

**DOI:** 10.1186/cc4904

**Published:** 2006-04-28

**Authors:** Wilson JQ Santos, Dirce MT Zanetta, Antonio C Pires, Suzana MA Lobo, Emerson Q Lima, Emmanuel A Burdmann

**Affiliations:** 1 Intensive Care Unit, Hospital de Base, São José do Rio Preto Medical School, São Paulo, Brazil; 2 Epidemiology Division, São José do Rio Preto Medical School, São Paulo, Brazil; 3 Endocrinology Division, São José do Rio Preto Medical School, São Paulo, Brazil; 4 Nephrology Division, São Jose do Rio Preto Medical School, São Paulo, Brazil

## Abstract

**Introduction:**

Acute tubular necrosis (ATN) is usually studied as a single entity, without distinguishing between ischaemic, nephrotoxic and mixed aetiologies. In the present study we evaluated the characteristics and outcomes of patients with ATN by aetiological group.

**Method:**

We conducted a retrospective comparison of clinical features, mortality rates and risk factors for mortality for the three types of ATN in patients admitted to the general intensive care unit of a university hospital between 1997 and 2000.

**Results:**

Of 593 patients with acute renal failure, 524 (88%) were classified as having ATN. Their mean age was 58 years, 68% were male and 52% were surgical patients. The overall mortality rate was 62%. A total of 265 patients (51%) had ischaemic ATN, 201 (38%) had mixed ATN, and 58 (11%) had nephrotoxic ATN. There were no differences among groups in terms of age, sex, APACHE II score and reason for ICU admission. Multiple organ failure was more frequent among patients with ischaemic (46%) and mixed ATN (55%) than in those with nephrotoxic ATN (7%; *P *< 0.0001). The complications of acute renal failure (such as, gastrointestinal bleeding, acidosis, oliguria and hypervolaemia) were more prevalent in ischaemic and mixed ATN patients. Mortality was higher for ischaemic (66%; *P *= 0.001) and mixed ATN (63%; *P *= 0.0001) than for nephrotoxic ATN (38%). When ischaemic ATN patients, mixed ATN patients and all patients combined were analyzed by multivariate logistic regression, the independent factors for mortality identified were different except for oliguria, which was the only variable universally associated with death (odds ratio [OR] 3.0, 95% confidence interval [CI] 1.64–5.49 [*P *= 0.0003] for ischaemic ATN; OR 1.96, 95% CI 1.04–3.68 [*P *= 0.036] for mixed ATN; and OR 2.53, 95% CI 1.60–3.76 [*P *< 0.001] for all patients combined]).

**Conclusion:**

The frequency of isolated nephrotoxic ATN was low, with ischaemic and mixed ATN accounting for almost 90% of cases. The three forms of ATN exhibited different clinical characteristics. Mortality was strikingly higher in ischaemic and mixed ATN than in nephrotoxic ATN. Although the type of ATN was not an independent predictor of death, the independent factors related to mortality were different for ischaemic, mixed and all patients combined. These data indicate that the three types of ATN represent different patient populations, which should be taken into consideration in future studies.

## Introduction

Acute renal failure (ARF) is frequent in intensive care units (ICUs), affecting up to 30% of patients [1-3]. It carries high morbidity, increases the length of hospital stay, increases hospital costs, is associated with high rates of mortality (60% or more) and is an independent risk factor for poor outcome in critically ill patients [1-4]. Acute tubular necrosis (ATN), diagnosis of which is usually based on clinical findings, is the most common cause of ARF in the hospital and in the ICU [3]. ATN may occur after ischaemic or nephrotoxic injury or after a combination of both (mixed ATN). Surprisingly, few studies have analyzed the three types of ATN separately [5,6], with almost all analyzing ATN as a single entity, without distinction between aetiologies [1,2,7]. Even recent consensus reports did not comment on the origin of ARF [8].

In the present study we evaluated a large cohort of ICU patients with a diagnosis of ATN, aiming to assess whether there were significant differences in demographic data, clinical picture and mortality between ischaemic, nephrotoxic and mixed ATN.

## Subjects and method

The present retrospective cohort study involved analysis of ICU patient files. The patients analyzed were older than 12 years and were hospitalized in the general ICU (24 beds) of a tertiary university hospital (700 beds) from January 1997 to January 2000. The protocol was approved by the local ethics committee.

### Participant selection

ARF was defined as a serum creatinine (SCr) of 1.8 mg/dl or more in patients with a SCr of 1.5 mg/dl or less during the 30 days preceding ICU admission. Patients who had a SCr above 1.5 mg/dl and no more than 4.0 mg/dl during the 30 days preceding ICU admission were viewed as having ARF if their SCr values had increased by 50% or more from baseline. Patients with a SCr of 1.8 mg/dl or greater but without known previous SCr were viewed as having ARF if their SCr normalized (≤1.5 mg/dl) or decreased at least 50% from its peak value during hospitalization. Patients with a SCr of 1.8 mg/dl or more, without known baseline SCr values and without SCr decrease, were viewed as having ARF only if history, renal ultrasound and laboratory examinations were indicative of this diagnosis.

### Definitions of acute tubular necrosis

Ischaemic ATN was defined as ARF resulting from situations causing inadequate renal blood flow during the 48 hours preceding the increase in SCr (volume depletion, heart failure, hypotension, shock, sepsis) without exposure to nephrotoxins. Nephrotoxic ATN was defined as ARF resulting from exposure to nephrotoxins during the 72 hours preceding the increase in SCr (radiocontrast medium, aminoglycoside, vancomycin, sulfamethoxazole, sulfadiazine, rifampicin, amphotericin B, cephalothin, cephalexin, acyclovir, foscarnet, pentamidine, zidovudine, indinavir, cyclosporine, tacrolimus, nonsteroidal anti-inflammatory drugs, angiotensin-converting enzyme inhibitors, angiotensin II receptor blockers, cisplatin, methotrexate, free myoglobin, free haemoglobin and increased serum bilirubin) without an ischaemic insult. Those who developed ARF after simultaneous ischaemic and nephrotoxic injuries were defined as having mixed ATN.

### Exclusion criteria

Patients were excluded if they had pre-renal ARF (defined as normalization or significant decrease in SCr over 24 hours after optimization of volume or heart function); post-renal ARF; or known or suspected diagnosis of vasculitis, glomerulonephritis, or acute interstitial nephritis. Patients were also excluded if they had a diagnosis of severe chronic renal failure (patients on chronic dialysis or with usual baseline SCr >4 mg/dl), if hospitalization time was under 24 hours, if they did not have previous SCr measurements and history, renal ultrasound and laboratory examinations did not allow a clear diagnosis of ARF, and if the patient files were incomplete.

### Characterization of the population and demographic data

The following data were recorded: age, sex, presence of a co-morbid condition, patient classification (medical or surgical), reason for ICU hospitalization, ICU hospitalization time (from ICU admission to ICU discharge or death), SCr concentration (admission, peak and discharge or death), admission APACHE II score and patient outcome.

### Complications of acute renal failure

The patients were screened for various potential complications developing after the diagnosis of acute renal failure (Table 1). The use of dialysis was also recorded.

### Other organ failures

Patients were analyzed for failure of other organs and systems developing at any time during their ICU stay, using the following definitions [9,10]. Respiratory failure was deemed to be present if there was a need for mechanical ventilation. Acute liver failure was defined as increased total bilirubin and/or prothrombin time greater than 60 s and/or International Normalized Ratio above 1.8 and/or hepatic encephalopathy developing up to 8 weeks after the beginning of liver disease associated with increased aspartate aminotransferase and alanine aminotransferase levels. Circulatory failure was defined as need for vasoactive drugs for maintenance of blood pressure. Central nervous system failure was considered to be present if the Glasgow Come Scale score was 8 or less. Finally, multiple organ failure was defined as simultaneous failure of three or more organs.

### Statistical analysis

Data are expressed as percentage, mean ± standard deviation, or median (range), as appropriate. When variables were normally distributed, one-way analysis of variance was performed to compare the groups; otherwise, the Kruskal-Wallis test was used. If the result was significant, the *post hoc *analysis with Bonferroni correction for multiple comparisons, Mann-Whitney *U *test, or χ^2 ^tests were conducted. Multivariate logistic regression was performed to evaluate risk factors for mortality associated with ATN. The independent variables were those significant at univariate analysis and those considered clinically important, controlling for potential confounding variables. The first model included age (reference: <60 years of age), number of co-morbid conditions (reference: none), APACHE II score (reference: <15), and the variables oliguria, sepsis, acidosis, hyperkalaemia, multiorgan failure, respiratory failure, shock and dialysis (using their absence as the reference). The model was first tested for ischaemic group and then a second analysis was performed for the mixed ATN group. Because of the relatively small number of patients with nephrotoxic ATN, we did not evaluate risk factors for mortality in this group by multivariate analysis. The third logistic regression analysis was performed with all three groups and also included the type of ATN (reference: nephrotoxic) as an independent variable in the first model. Backward variable selection was used serially to remove nonsignificant factors. The variables that, when excluded, introduced a change in parameter estimates greater than 10% were re-introduced to the model to account for confounding. Goodness-of-fit of the model was assessed using the Hosmer and Lemeshow test. Wald test was used to assess the significance of variables in the models. *P *< 0.05 was considered statistically significant. For multiple comparisons with Bonferroni correction, *P *< 0.017 was considered statistically significant. The data were analyzed using EPI-Info (version 6.04; Centers for Diseases Control and Prevention, Atlanta, GA, USA; 2001) and BMDP (version PC90 [1990 IBM PC/MS-DOS]; BMDPRL Statistical Software, Los Angeles, CA, USA).

## Results

During the period analyzed, 3,676 patients were admitted to the ICU. Among them 832 had a SCr of 1.8 mg/dl or greater. A total of 308 patients were excluded (11 with post-renal ARF, 14 with a known or suspected diagnosis of vasculitis, glomerulonephritis, or acute interstitial nephritis, 36 with hospitalization time <24 hours, 44 with pre-renal ARF, 47 with incomplete files and 156 with severe chronic renal failure). In all, 524 patients with ATN (14.2% of all ICU patients evaluated) were included in the study. Among these, 50.9% (*n *= 267) developed ARF in the ICU whereas 49.1% were admitted with already increased SCr.

### Characterization of the overall population

The mean age of the patients was 58 ± 19 years (seven patients were <18 years of age: one was 12 years old, one was 13, one was 14, two were 15 and two were 17). Sixty-eight percent of the patients were male, 52% patients were surgical, the mean APACHE II score was 20.8 ± 7.4 and hospitalization time was 7 days (range 2 to 147 days). Dialysis was used in 11.7% of the patients, and the hospital mortality rate was 61.5%.

The peak SCr was 3.25 ± 1.51 mg/dl and the mean SCr at death or discharge was 2.64 ± 1.60 mg/dl. Hyperkalaemia developed in 26.3% of the patients, hypervolaemia in 13.4%, and 13.4% suffered a gastrointestinal bleeding. The majority of the patients presented with infection (61.3%) and hypotension (89.9%).

When patients were divided according to type of ATN, it was found that 265 (51%) had ischaemic ATN, 201 (38%) had mixed ATN and 58 (11%) had nephrotoxic ATN.

### Comparisons among the three acute tubular necrosis groups

#### Age, sex, APACHE II score and patient classification

Age, sex and APACHE II score were similar among the three groups. There was a higher number of medical patients in the ischaemic group than in the mixed group (54% versus 41%; *P *< 0.017). These data are summarized in Table 2.

#### Comorbid conditions

More patients in the ischaemic group than in the mixed group had at least one co-morbid condition (62% versus 49%; *P *< 0.01). When co-morbid condition were analyzed individually, a greater frequency of hypertension was observed in the nephrotoxic group than in the ischaemic (36% versus 21%; *P *= 0.02) and mixed groups (18%; *P *< 0.01). There were no differences among the three groups with respect to pulmonary, hepatic, or cardiovascular co-morbid conditions (Table 2).

#### Reason for intensive care unit admission

The reasons for ICU admission were similar in the three groups, with postoperative patients and those with infection dominating, followed by several other causes (Table 3).

#### Complications of acute renal failure

Oliguria was more frequent in the ischaemic (49%) and mixed (58%) ATN groups than in the nephrotoxic ATN group (38%). However, the difference was statistically significant only between mixed and nephrotoxic ATN groups (*P *= 0.01).

Gastrointestinal bleeding was more frequent in the ischaemic (17%) and mixed (12%) ATN groups than in the nephrotoxic ATN group (2%; *P *< 0.01 versus ischaemic group).

Infection was more frequent in the mixed ATN group (74%) than in the ischaemic (54%; *P *< 0.01) and nephrotoxic (53%; *P *< 0.0001) ATN groups.

Hypervolaemia was more prevalent in the mixed (20%) and ischaemic (14%) ATN groups than in the nephrotoxic group, although this finding was not statistically significant.

Metabolic acidosis was more frequent in the ischaemic (73%) and mixed (81%) ATN groups than in the nephrotoxic ATN group (64%; *P *= 0.01 versus mixed ATN). There were no statistically significant differences among groups with respect to the percentage of patients with hyperkalaemia or hyponatraemia.

On ICU admission, SCr was 1.98 ± 0.88 mg/dl in the ischaemic ATN group, 1.81 ± 0.88 mg/dl in the mixed ATN group and 1.63 ± 0.85 mg/dl in the nephrotoxic ATN group (*P *= 0.003 versus ischaemic ATN). Peak SCr was higher in the ischaemic (3.24 ± 1.59 mg/dl) and in the mixed (3.41 ± 1.5 mg/dl) ATN groups than in the nephrotoxic ATN group (2.78 ± 1.03; *P *< 0.01 versus mixed ATN). The discharge SCr was also higher in the ischaemic (2.70 ± 1.47 mg/dl) and mixed (2.98 ± 1.56 mg/dl) ATN groups than in the nephrotoxic ATN group (1.96 ± 0.96 mg/dl; *P *< 0.01 versus mixed ATN).

More patients in the mixed ATN group than in the ischaemic and nephrotoxic ATN groups underwent dialysis (17%, 9% [*P *= 0.01 versus mixed] and 7%, respectively).

Data on the patients' clinical picture are summarized in Table 4.

#### Hospitalization time

Hospitalization time was more prolonged in mixed (10 days, range 2 to 147 days) and nephrotoxic (8.5 days, range 2 to 49 days) ATN groups than in the ischaemic ATN group (5 days, range 2 to 69 days; *P *< 0.0001 versus mixed and *P *= 0.001 versus nephrotoxic).

#### Multiple organ failure

The ischaemic and mixed ATN groups had a higher frequency of multiple organ failure in comparison with the nephrotoxic ATN group (46% and 55%, respectively, versus 7%; *P *< 0.0001 for both).

There were more patients with respiratory failure in the ischaemic (87%; *P *< 0.01 versus nephrotoxic) and mixed (96%; *P *< 0.0001 versus nephrotoxic) ATN groups than in the nephrotoxic ATN group (69%). There were significantly more patients with respiratory failure in the mixed ATN group than in the ischaemic ATN group (*P *= 0.001).

There was a higher frequency of shock in the ischaemic and mixed ATN groups than in the nephrotoxic ATN group (83% and 87%, respectively, versus 14%; *P *< 0.0001 for both).

The ischaemic and mixed ATN groups included more patients with hepatic failure than did the nephrotoxic ATN group (14% and 8%, respectively, versus 3%), but this difference was not statistically significant.

In the same way, the ischaemic and mixed ATN groups included comatose patients than did the nephrotoxic group (40% and 34%, respectively, versus 21%), but the difference was statistically significant only for ischaemic versus nephrotoxic ATN groups (*P *= 0.01).

These data are summarized in Table 4.

#### Mortality

Mortality was almost twofold higher in ischaemic (66%) and mixed (63%) ATN patients than in the nephrotoxic ATN population (38%; *P *= 0.001 versus ischaemic and *P *= 0.0001 versus mixed). Logistic regression models were constructed to evaluate risk factors for death. The first and second analyses included the ischaemic and mixed ATN groups, respectively. The third analysis included all patients from the three groups. The only variable universally related to death in the three analyses was oliguria. The significant variables in the final models are listed in Table 5.

## Discussion

During the past few decades our understanding of the mechanisms involved in the development and maintenance of experimental ARF has advanced considerably. However, little has been integrated into clinical practice to prevent, treat, or accelerate recovery of renal function in patients with ARF. In fact, the mortality rate of patients with ARF remains high, and can exceed 60% when only ICU patients are analyzed [1-3].

The nature and severity of the factors that trigger renal failure may partly be responsible for maintaining this increased mortality rate. Nephrotoxic ARF, which is more prevalent in patients hospitalized in medical wards, is associated with lower mortality than ARF of ischaemic aetiology [3,6,11]. However, few studies have analyzed the impact that the triggering factor (ischaemic or nephrotoxic) has on ARF mortality, or whether the prognostic factors and characteristics differ among patients with ARF from ischaemic, mixed and nephrotoxic etiology [6].

In the present study the 524 ICU patients with ARF presented with characteristics similar to those previously reported by other investigators (such as, advanced age, higher proportion of males, presence of multiple organ failure and high mortality rate [62%]). The distribution of the different types of ATN was also similar to those in other studies reporting frequency of ischaemic and mixed ATN [1,6,7,12]. In fact, other studies conducted in ICU patients, both retrospective and prospective, have demonstrated similar trends toward a greater frequency of multifactorial aetiology and reduced incidence of isolated nephrotoxicity as the cause of ATN [1,7,12-14]. However, we found mortality rates in patients with ischaemic (66%) and mixed (63%) ARF to be almost twice the rate in patients with nephrotoxic ARF (38%). Weisberg and coworkers [6] analyzed the placebo group in the Auriculin Anaritide Acute Renal Failure Study and, consistent with the findings of the present study, reported mortality rates in patients with ischaemic and mixed ARF to be three times the rate in patients with nephrotoxic ARF. It is conceivable that ischaemia is a determining factor in mortality rates in patients with ARF. Several prospective studies have demonstrated the impact of ischaemic factors (hypotension, shock, use of vasoactive drugs, sepsis) on mortality rates in patients with ARF [1,2,5]. By means of a prognostic score (Acute Tubular Necrosis-Individual Severity Index [ATN-ISI]), Liaño and coworkers [5] clearly demonstrated the protective effect of nephrotoxicity and the negative impact of ischaemia on mortality rates in this population.

When all patients were analyzed together by logistic regression, we found that the type of ATN was not an independent risk factor for death. Hence, the differences in mortality rates found between the three ATN groups are probably related to patient characteristics in each group, with a higher frequency of factors negatively affecting prognosis among patients with ischaemic and mixed ATN. There was no difference among groups in reason for ICU admission or APACHE II score. However, the frequency of multiple organ failure, especially involving cardiovascular, respiratory and neurological systems, was higher in patients with ischaemic and mixed ATN. Similarly, complications resulting from renal failure (for example, gastrointestinal bleeding, acidosis, oliguria and hypervolaemia) were more common in these patients. The finding that there were different independent risk factors for death in the ischaemic and mixed groups and for all patients combined supports the suggestion that the patients in the three groups differed. In the study conducted by Weisberg and coworkers [6] respiratory and liver failures were more prevalent in patients with ischaemic ATN, and there was no difference between groups with respect to oliguria.

Oliguria is among the major prognostic factors for mortality in ARF [1,5,7,15-17], but only one study [6] analyzed its frequency according to type of ATN. Weisberg and coworkers [6] did not identify any differences in the frequency of oliguria among patients with ischaemic (26%) and nephrotoxic ATN (25%). In the present study the frequency of oliguria was higher among patients with ischaemic and mixed ATN. However, on univariate analysis there was a statistically significant difference only for the mixed and nephrotoxic group. In prognostic scores specific for ARF patients, especially those by Liaño [5] and Mehta [7] and their coworkers, the importance of diuresis in these multivariate mortality predictive models is clear. The impact of diuresis on outcome in patients with ARF is better characterized when diuresis is analyzed as a continuous variable and not as a categorical variable (oliguric versus not oliguric) [7,15]. Confirming the importance of diuresis in determining outcome among ARF patients, oliguria was the only common prognostic factor for mortality in ischaemic and mixed ATN and for all patients combined in the multivariate logistic regression analysis.

The difference in mortality rates between different types of ATN might also be influenced by the presence of co-morbidity. When analyzed individually there were no differences in rates of co-morbidities between the three types of ATN, except for a higher frequency of arterial hypertension in patients with nephrotoxic ATN. In contrast, the proportions of patients with one or more co-morbid condition were higher among the ischaemic and nephrotoxic ATN groups. The presence of at least one co-morbid condition was an independent risk factor for death when the entire population and mixed ATN patients were analyzed but not for ischaemic ATN. In an observational study of 306 critically ill ARF patients [18], the presence of a co-morbid condition at ICU admission was the only independent risk factor for mortality. On the other hand, even after adjustment for differences in co-morbidities, Levy and coworkers [19] observed that patients with ARF had greater mortality than did those who did not have renal failure after infusion of iodated contrast. Therefore, this difference in mortality between the different types of ATN probably not only results from previous health status but also from the complications caused by the initial insult.

Few patients underwent dialysis in the present study, similar to the frequency of dialysis (11%) reported by Clermont and coworkers [20] in a recent analysis of ARF in the ICU. One possible explanation for this is that a relatively low creatinine threshold was used in the definition of ARF; in comparison with studies using a creatinine of 3 mg/dl or more for diagnosis of ARF [1], which included a higher proportion of dialyzed patients. This might have allowed inclusion of patients with ARF of comparatively lower severity in the present study. Indeed, in the present study peak creatinine for the three groups was below 4 mg/dl and, consistent with this, there was a relatively small percentage of patients with classic indications for dialysis, such as hyperkalaemia, hypervolaemia and bleeding. Although the institutional protocol did not impose any limitation on the use of renal replacement therapy (RRT) when indicated, at the time of the present analysis haemofiltration was rarely performed in our institution because of the high cost of this treatment. This might have limited the use of dialysis in patients with severe haemodynamic instability, which was prevalent in both ischaemic and mixed groups. When dialyzed and nondialyzed patients were compared, the mortality rate was slightly higher in the former group, although this finding was not statistically significant (70.4% in dialyzed versus 60.05% in nondialyzed patients; data not included under Results, above). The types of RRTs used included intermittent haemodialysis, slow low-efficiency haemodialysis and peritoneal dialysis. It is unlikely that this aspect of practice influenced the outcome of the studied population. Several studies failed to show increments in patient survival when continuous RRT was compared with intermittent or hybrid RRT [21-23]. Furthermore, the influence of peritoneal dialysis on ARF prognosis is controversial [24,25]. Another possible explanation for the low rate of dialysis is that, because of the critical condition of the patients (APACHE II scores >20), the nephrologist was called late or not even called at all because of 'do no resuscitate' orders. It is interesting that the ICU stay of dialyzed patients was 20.4 days as compared with 10.8 days in the nondialyzed group (data not included under results). Thus, it is conceivable that most of the patients died before they dialysis was indicated.

## Conclusion

This study showed that there are marked differences in clinical characteristics between the three types of ATN. Ischaemic and mixed ATN were associated with higher frequencies of multiple organ failure and complications of ARF. Mortality rates were clearly higher with ischaemic and mixed ATN than with nephrotoxic ATN. Although the type of ATN was not an independent risk factor for death, the ischaemic group, mixed group and all patients combined had different independent risk factors for mortality. The only independent prognostic factor for mortality common to the three groups was oliguria.

These findings suggest that ATN patients should not be analyzed as a single population. Such simplistic analysis might have influenced the results of clinical trials that did not yield the expected results in ARF patients.

## Key messages

• 	Isolated nephrotoxicity-induced ATN was infrequent in ICU patients. Almost 90% of them had ischaemia, isolated or combined with nephrotoxicity, as etiologic factor for ATN.

• 	The three etiologies of ATN, ischaemic, mixed and nephrotoxic had different clinical pictures.

• 	The independent factors related to mortality were not the same for ischaemic, mixed and all patients population.

• 	ATN of ischaemic and mixed etiology had a striking elevated mortality as compared to nephrotoxic ATN.

• 	These data indicated that ischaemic, mixed and nephrotoxic ATN are composed by different populations, and suggests that the type of ATN should be taken in consideration in acute renal failure studies

## Abbreviations

APACHE = Acute Physiology and Chronic Health Evaluation; ARF = acute renal failure; ATN = acute tubular necrosis; ICU = intensive care unit; RRT = renal replacement therapy; SCr = serum creatinine.

## Competing interests

The authors declare that they have no competing interests.

## Authors' contributions

All authors made substantial contribution to the study design and methods. DMTZ specifically contributed to the statistical methods and power calculations. EAB, EQL and WJQS drafted the manuscript and all other authors critically revised it for important intellectual content. All authors read and approved the final version of the manuscript.

## Figures and Tables

**Table 1:** Complications of acute renal failure

Complication	Details/comments
Presence of coma	Glasgow Coma Scale score ≤8 without sedative drugs
Bleeding	Presence of active bleeding with decrease in haematocrit
Shock	Need for vasoactive drugs for blood pressure maintenance
Hypertension	systolic blood pressure >140 mmHg and/or diastolic blood pressure >90 mmHg
Oliguria	Diuresis <20 ml/hour or <400 ml/day
Hypervolaemia	Oedema plus hypertension and/or left ventricular failure and/or acute pulmonary oedema
Hyperkalaemia	Serum potassium >5.5 mEq/l on at least two consecutive measurements
Hyponatraemia	Serum sodium <130 mEq/l on at least two consecutive measurements
Metabolic acidosis	Blood pH <7.20 and/or serum bicarbonate <20 mEq/l on at least two consecutives measurements
Infection developing after the diagnosis of acute tubular necrosis	White blood cell count >15,000/mm^3 ^or <4,000/mm^3 ^(in the absence of haematological disease), axillary temperature ≥37.8°C or <36°C and heart rate >90 beats/minute, in the presence of an infectious site demonstrated by radiography, urinalysis, cerebrospinal fluid examination, ultrasound, or positive cultures

**Table 2:** Demographic data, according to cause of acute tubular necrosis

Characteristic	Cause of ATN		
	
	Ischaemic (*n *= 265)	Mixed (*n *= 201)	Nephrotoxic (*n *= 58)
Age (years)	56.7 ± 18.8	58.8 ± 18.3	58.9 ± 20.1
Sex			
Male	188 (71%)	133 (66%)	40 (69%)
Female	77 (29%)	68 (34%)	18 (31%)
Medical	143 (54%)*	82 (41%)	28 (48%)
Surgical	122 (46%)	119 (59%)	30 (52%)
APACHE II score	21.0 ± 7.3	20.7 ± 7.2	20.3 ± 8.4
Co-morbid conditions			
Hypertension	59 (21%)	36 (18%)	21 (36%)^†^
Pulmonary diseases	17 (6.4%)	14 (7%)	3 (5.2%)
Liver diseases	34 (12.8%)	16 (8%)	2 (3.4%)
Cardiovascular diseases	44 (16.6%)	26 (12.9%)	7 (12.1%)
At least one co-morbid condition	164 (62%)*	98 (49%)	38 (65%)

Data are expressed as mean ± standard deviation or *n *(%). *P < 0.017, ischaemic versus mixed acute tubular necrosis (ATN); ^†^*P *< 0.017, mixed versus nephrotoxic ATN.

**Table 3:** Intensive care unit admission diagnoses according to cause of acute tubular necrosis

Diagnosis/cause of admission	Cause of acute tubular necrosis		
	
	Ischaemic (*n *= 265)	Mixed (*n *= 201)	Nephrotoxic (*n *= 58)
Postoperative	80 (30.2%)	81 (40.3%)	24 (41.4%)
Infection	45 (17%)	45 (22.4%)	14 (24.1%)
Trauma	17 (6.4%)	12 (6%)	3 (5.2%)
Neurological	18 (6.8%)	5 (2.5%)	5 (8.6%)
Shock	15 (5.7%)	7 (3.5%)	1 (1.7%)
Gastrointestinal bleeding	20 (7.5%)	17 (8.5%)	0 (0%)
After cardiac arrest	15 (5.7%)	8 (4%)	2 (3.4%)
Pancreatitis	11 (4.2%)	4 (2%)	1 (1.7%)
Cardiac failure	9 (3.4%)	4 (2%)	0 (0%)
Acute respiratory failure	12 (4.5%)	6 (3%)	4 (6.9%)
Metabolic disorders	9 (3.3%)	4 (2%)	0 (0%)
Other	14 (5.3%)	8 (4%)	4 (6.9%)

Data are expressed as *n *(%).

**Table 4:** Clinical features of patients according to cause of acute tubular necrosis

Clinical feature	Cause of acute tubular necrosis		
	
	Ischaemic (*n *= 265)	Mixed (*n *= 201)	Nephrotoxic (*n *= 58)
Multiorgan failure	122 (46%)^†^	110 (55%)^‡^	4 (7%)
Respiratory failure	230 (87%)^†^	193 (96%)^‡*^	40 (69%)
Shock	220 (83%)^†^	175 (87%)^‡^	8 (14%)
Hepatic failure	37 (14%)^†^	16 (8%)	2 (3%)
Coma	106 (40%)^†^	68 (34%)	12 (21%)
Infection	143 (54%)	149 (74%)^‡*^	31 (53%)
Gastrointestinal bleeding	45 (17%)^†^	24 (12%)	4 (7%)
Oliguria	130 (49%)	116 (58%)^‡^	22 (38%)
Hypervolaemia	37 (14%)	40 (20%)	6 (10%)
Acidosis	193 (73%)	163 (81%)^‡^	37 (64%)
Admission creatinine (mg/dl)	1.98 ± 0.88^†^	1.81 ± 0.88	1.63 ± 0.85
Peak creatinine (mg/dl)	3.24 ± 1.59	3.41 ± 1.50^‡^	2.78 ± 1.03
Discharge creatinine (mg/dl)	2.70 ± 1.47	2.98 ± 1.56*	1.96 ± 0.96
Dialysis	24 (9%)	34 (17%)*	4 (7%)
Mortality	175 (66%)^†^	127 (63%)^‡^	22 (38%)

Data are expressed as mean ± standard deviation or as *n *(%). *P < 0.017 ischaemic versus mixed acute tubular necrosis ATN; ^†^*P *< 0.017 ischaemic versus nephrotoxic ATN; ^‡^*P *< 0.017 mixed versus nephrotoxic ATN.

**Table 5:** Logistic regression analysis for mortality risk factors according to cause of acute tubular necrosis

Cause of ATN	Factor	OR (95% CI)	*P*
Ischaemic	Oliguria	3.00 (1.64–5.49)	0.0003
	Acidosis	2.14 (1.09–4.19)	0.03
	Hyperkalaemia	2.64 (1.2–5.81)	0.015
	Multiple organ failure	2.67 (1.35–5.25)	0.005
Mixed	Oliguria	1.96 (1.04–3.68)	0.036
	One co-morbid condition or more	2.04 (1.09–3.83)	0.024
Ischaemic, mixed and nephrotoxic ATN (all patients)	Oliguria	2.53 (1.68–3.76)	<0.001
	One co-morbid condition or more	1.88 (1.24–2.84)	0.002
	Acidosis	1.90 (1.20–3.04)	0.003
	Multiple organ failure	1.90 (1.21–3.02)	0.003
	Respiratory insufficiency	2.40 (1.23–4.63)	0.005
	Shock	2.29 (1.30–4.08)	0.002

ATN, acute tubular necrosis; CI, confidence interval; OR, odds ratio.

## References

[B1] Brivet FG, Kleinknecht DJ, Loirat P, Landais PJ (1996). Acute renal failure in intensive care units -- causes, outcome, and prognostic factors of hospital mortality; a prospective, multicenter study. French Study Group on Acute Renal Failure. Crit Care Med.

[B2] de Mendonca A, Vincent JL, Suter PM, Moreno R, Dearden NM, Antonelli M, Takala J, Sprung C, Cantraine F (2000). Acute renal failure in the ICU: risk factors and outcome evaluated by the SOFA score. Intensive Care Med.

[B3] Liano F, Junco E, Pascual J, Madero R, Verde E (1998). The spectrum of acute renal failure in the intensive care unit compared with that seen in other settings. The Madrid Acute Renal Failure Study Group. Kidney Int Suppl.

[B4] Metnitz PG, Krenn CG, Steltzer H, Lang T, Ploder J, Lenz K, Le Gall JR, Druml W (2002). Effect of acute renal failure requiring renal replacement therapy on outcome in critically ill patients. Crit Care Med.

[B5] Liano F, Gallego A, Pascual J, Garcia-Martin F, Teruel JL, Marcen R, Orofino L, Orte L, Rivera M, Gallego N (1993). Prognosis of acute tubular necrosis: an extended prospectively contrasted study. Nephron.

[B6] Weisberg LS, Allgren RL, Genter FC, Kurnik BR (1997). Cause of acute tubular necrosis affects its prognosis. The Auriculin Anaritide Acute Renal Failure Study Group. Arch Intern Med.

[B7] Mehta RL, Pascual MT, Gruta CG, Zhuang S, Chertow GM (2002). Refining predictive models in critically ill patients with acute renal failure. J Am Soc Nephrol.

[B8] Bellomo R, Ronco C, Kellum JA, Mehta RL, Palevsky P, Acute Dialysis Quality Initiative workgroup (2004). Acute renal failure: definition, outcome measures, animal models, fluid therapy and information technology needs: the Second International Consensus Conference of the Acute Dialysis Quality Initiative (ADQI) Group. Crit Care.

[B9] Le Gall JR, Klar J, Lemeshow S, Saulnier F, Alberti C, Artigas A, Teres D (1996). The Logistic Organ Dysfunction system. A new way to assess organ dysfunction in the intensive care unit. ICU Scoring Group. JAMA.

[B10] Vincent JL, Moreno R, Takala J, Willatts S, De Mendonca A, Bruining H, Reinhart CK, Suter PM, Thijs LG (1996). The SOFA (Sepsis-related Organ Failure Assessment) score to describe organ dysfunction/failure. On behalf of the Working Group on Sepsis-Related Problems of the European Society of Intensive Care Medicine. Intensive Care Med.

[B11] Barretti P, Soares VA (1997). Acute renal failure: clinical outcome and causes of death. Ren Fail.

[B12] Cole L, Bellomo R, Silvester W, Reeves JH (2000). A prospective, multicenter study of the epidemiology, management, and outcome of severe acute renal failure in a 'closed' ICU system. Am J Respir Crit Care Med.

[B13] Chertow GM, Christiansen CL, Cleary PD, Munro C, Lazarus JM (1995). Prognostic stratification in critically ill patients with acute renal failure requiring dialysis. Arch Intern Med.

[B14] Douma CE, Redekop WK, van der Meulen JH, van Olden RW, Haeck J, Struijk DG, Krediet RT (1997). Predicting mortality in intensive care patients with acute renal failure treated with dialysis. J Am Soc Nephrol.

[B15] Burdmann EA, Yu L, Molitoris BA, Finn WF (2001). Metabolic and electrolyte disturbances: secondary manifestations. Acute Renal Failure: a Companion to Brenner & Rector's The Kidney.

[B16] Chertow GM, Lazarus JM, Paganini EP, Allgren RL, Lafayette RA, Sayegh MH (1998). Predictors of mortality and the provision of dialysis in patients with acute tubular necrosis. The Auriculin Anaritide Acute Renal Failure Study Group. J Am Soc Nephrol.

[B17] Neveu H, Kleinknecht D, Brivet F, Loirat P, Landais P (1996). Prognostic factors in acute renal failure due to sepsis. Results of a prospective multicentre study. The French Study Group on Acute Renal Failure. Nephrol Dial Transplant.

[B18] Schroeder TH, Hansen M, Dinkelaker K, Krueger WA, Nohe B, Fretschner R, Unertl K (2004). Influence of underlying disease on the outcome of critically ill patients with acute renal failure. Eur J Anaesthesiol.

[B19] Levy EM, Viscoli CM, Horwitz RI (1996). The effect of acute renal failure on mortality. A cohort analysis. JAMA.

[B20] Clermont G, Acker C, Angus DC, Sirio CA, Pinsky MR, Johnson JP (2002). Renal failure in the ICU: comparison of the impact of acute renal failure and end-stage renal disease on ICU outcomes. Kidney Int.

[B21] Guerin C, Girard R, Selli JM, Ayzac L (2002). Intermittent versus continuous renal replacement therapy for acute renal failure in intensive care units: results from a multicenter prospective epidemiological survey. Intensive Care Med.

[B22] Mehta RL, McDonald B, Gabbai FB, Pahl M, Pascual MT, Farkas A, Kaplan RM, Collaborative Group for Treatment of ARF in the ICU (2001). A randomized clinical trial of continuous versus intermittent dialysis for acute renal failure. Kidney Int.

[B23] Uehlinger DE, Jakob SM, Ferrari P, Eichelberger M, Huynh-Do U, Marti HP, Mohaupt MG, Vogt B, Rothen HU, Regli B (2005). Comparison of continuous and intermittent renal replacement therapy for acute renal failure. Nephrol Dial Transplant.

[B24] Phu NH, Hien TT, Mai NT, Chau TT, Chuong LV, Loc PP, Winearls C, Farrar J, White N, Day N (2002). Hemofiltration and peritoneal dialysis in infection-associated acute renal failure in Vietnam. N Engl J Med.

[B25] Chitalia VC, Almeida AF, Rai H, Bapat M, Chitalia KV, Acharya VN, Khanna R (2002). Is peritoneal dialysis adequate for hypercatabolic acute renal failure in developing countries?. Kidney Int.

